# Treatment of Cystic Goitre by Injection

**Published:** 1874-10

**Authors:** J. Ewing Mears


TREATMENT OF CYSTIC GOITRE BY EVACUATION AND INJECTION OF THE SOLUTION OF THE PERCHLORIDE OF IRON—*By J. Ewing Mears, M.D.*—In the London *Lancet* of May 11, 1872, Dr. Morell Mackenzie reported a number of cases of cystic bronchocele which he had treated with eminent success by tapping and injecting with the solution of the perchloride of iron. As stated in the article, the cysts, by this method of treatment, were converted into chronic abscesses, and it was only necessary to conduct these to a termination in order to effect the cure of the bronchocele. The operation is exceedingly simple, and is described as follows: The cyst is first emptied, the trocar being introduced at its most dependent point, through the canula, which is allowed to remain; a drachm or more (the quantity being determined by the size of the cyst) of the solution of the perchloride of iron is injected, and the opening of the canula closed by a piece of cork or wood, cut to the proper size. The solution of iron is permitted to remain in the cyst for three or four days, according to the degree of inflammation which it is thought necessary to produce. At the end of the prescribed time it is withdrawn, the canula, with the opening closed, being retained in position. Poultices of linseed meal are now applied over the cyst, and when suppuration is fully established the plug in the canula is removed, and free drainage is secured. The canula is not removed until the discharge is limited in amount, and its consistence such as to permit its easy exit through the wound. The duration of treatment was reported to vary from three weeks to four months.

Having under my care, at the time of reading this article, a patient who was suffering from bronchocele, I determined to treat it according to the plan so successfully employed by Dr. Mackenzie. Although I was not able, as will be seen, to follow to the letter the instructions given, still the success was complete, and I feel it a duty to report the case which so entirely confirms the plan of Dr. Mackenzie. It gives to the surgeon a method of treatment in these cases which is at once simple and devoid of danger.

The patient, a female, aged twenty-eight years, first noticed the tumor in the neck some twelve years ago. Its growth had been very slow, and for a period of four years it seemed to remain stationary. During the last year it had enlarged in size until it produced quite a deformity, and at times interfered with swallowing. It was, at the time of the operation, the size of a large-sized hen's egg, being developed rather more to the left of the median line of the neck. It was freely movable, rising and falling with the movements of the larynx and trachea in deglutition. Various plans of treatment had been employed to effect its removal. I had already tried simple tapping and the internal administration of sorbefacient remedies, with also local applications.

Owing to the failure to obtain the proper form of trocar and canula, I was unable to secure the latter in the cyst after I had tapped it, and injected a drachm of the solution of the perchloride of iron. The injection was, however, entirely retained by the closure of the puncture made by the small trocar. On the third day symptoms of inflammation appeared, and the neck was quite swollen; slight febrile movement was also present. On the fourth day I reopened the cyst, from which there escaped a small quantity of a viscid, tarry substance. Poultices were now applied, and in a few days suppuration was established, the pus escaping through the puncture, which was kept open by the use of the probe. In six weeks the discharge ceased and the opening closed, leaving but a slight swelling over the site of the tumor. Three months after, when I saw the patient, this swelling had disappeared, and a small cicatrix marked the position of the cyst. *Philadelphia Medical Times—August.*

